# Food

**Published:** 1893-12

**Authors:** 


### EDITORIAL.

### Food.

The question of food, both as to variety and purity, is one now attracting more attention than ever before. As the subject of nutrition in physiological research is becoming better understood, the nature of the material to supply the nutrient demand is becoming more and more a question for thorough investigation.

It is now, better than ever before, understood that some substances used as food answer well the demand of nature, while others are in a more or less marked degree inefficient in this respect, and many substances are so entirely devoid of the nutrient material for the animal economy as to render them unfit for use. This question, however, we do not now propose to consider, but will direct attention in a few words to the purity of foods.

Much attention is being given in this direction, because that through the cupidity and avarice of manufacturers and dealers many of our best articles of food have been grossly adulterated, having mixed with them, oftentimes in large quantities, material of little or no nutrient qualities, and in other instances, substances of a pernicious and sometimes a poisonous character.

Many dealers in foods are conscientious in securing and dispensing pure articles, but even such persons are oftentimes imposed upon by unscrupulous dealers or manufacturers. Public attention is being so much awakened to this subject that stringent laws regulating it are urgently demanded in many quarters. It is true, that laws already exist, the intent of which is to regulate this matter, but they are of such character as in many particulars to render them difficult of execution.

Such laws should be clear and explicit, with such penalties as to deter offenders in this line. Very stringent and efficient laws have been enacted regulating the dispensing of drugs, and it is equally if not more important that our food products should be thoroughly guarded, so that the people may feel a security in this matter.

Food is in constant demand by every individual of every community. Medicines are in demand by comparatively few, and usually only at long intervals.

For the purpose of further enlisting the public interest to the importance of the subject, a Pure Food Exposition is to be held in Cincinnati, at Music Hall, beginning February 26, 1894, which will continue until March 19. In addition to the main display of foods of all kinds, there will be cooking contests, lectures on the art, the latest devices for cooking by electricity, and the preparation of foods generally.

This exposition will be one of very great interest and value to the people not only of this vicinity but throughout the country, as it is a national affair. Many lessons will be learned that will promote the health of the people of the present and the future. New articles of food will be presented, and new and valuable methods of preparation for a far larger number will also attract attention. There will be presented lectures on every branch of the subject, and in addition object lessons, which should be made available by individuals from every portion of our country.

This is a subject to which the special attention of every one having charge of the general health of the people should be given. It is a question, however, of universal interest, and we trust the occasion will receive the attention that its great importance requires.

